# Effects of Etanercept against Transient Cerebral Ischemia in Diabetic Rats

**DOI:** 10.1155/2015/189292

**Published:** 2015-11-19

**Authors:** Naohiro Iwata, Hiroko Takayama, Meiyan Xuan, Shinya Kamiuchi, Hirokazu Matsuzaki, Mari Okazaki, Yasuhide Hibino

**Affiliations:** ^1^Laboratory of Immunobiochemistry, Department of Clinical Dietetics & Human Nutrition, Faculty of Pharmaceutical Sciences, Josai University, Saitama 350-0295, Japan; ^2^Laboratory of Organic and Medicinal Chemistry, School of Pharmaceutical Sciences, Faculty of Pharmaceutical Sciences, Josai University, Saitama 350-0295, Japan; ^3^Laboratory of Pharmacology, School of Pharmaceutical Sciences, Faculty of Pharmaceutical Sciences, Josai University, Saitama 350-0295, Japan

## Abstract

Diabetes mellitus is known to exacerbate acute cerebral ischemic injury. Previous studies have demonstrated that infarction volumes caused by transient cerebral ischemia were greater in diabetic rats than in nondiabetic rats. Tumor necrosis factor-*α* (TNF-*α*) is a proinflammatory protein produced in the brain in response to cerebral ischemia that promotes apoptosis. Etanercept (ETN), a recombinant TNF receptor (p75)-Fc fusion protein, competitively inhibits TNF-*α*. Therefore, we evaluated the neuroprotective effects of chronic or acute treatment with ETN on cerebral injury caused by middle cerebral artery occlusion/reperfusion (MCAO/Re) in rats with streptozotocin-induced diabetes. Furthermore, we evaluated the effects of ETN against the apoptosis and myeloperoxidase activity. Single administration of ETN before MCAO significantly suppressed exacerbation of cerebral damage in nondiabetic rats, as assessed by infarct volume. In contrast, the diabetic state markedly aggravated MCAO/Re-induced cerebral damage despite ETN treatment within 24 h before MCAO. However, the damage was improved by repeated administration of ETN at 900 *μ*g/kg/daily in rats in an induced diabetic state. These results suggested that repeated administration of ETN can prevent exacerbation of cerebral ischemic injury in the diabetic state and is mainly attributed to anti-inflammatory effects.

## 1. Introduction

Diabetes mellitus (DM) is a metabolic disorder associated with chronic hyperglycemia, which is known to enhance systemic oxidative stress, predisposing patients to diabetic complications. World Health Organization data show that approximately 386 million people worldwide are currently suffering from diabetes, which is a major risk factor for atherosclerotic diseases, such as acute brain ischemia [1, 2]. Moreover, diabetic patients have a higher risk of stroke than nondiabetic patients and are more likely to have a poor prognosis and increased mortality after stroke [3, 4]. Previous studies have demonstrated that diabetes increased oxidative stress and inflammation in the brain [5] and aggravated cerebral ischemic injury in animal models [6–8]. Brain injury induced by focal ischemia is characterized by significant and rapid upregulation of cytokines, such as tumor necrosis factor-*α* (TNF-*α*). It is well known that inflammation has an essential role in the pathogenesis of transient cerebral ischemic injury [9].

TNF-*α* is a proinflammatory cytokine produced not only by macrophages but also by a broad variety of other cell types, such as endothelial cells, adipose tissue, fibroblasts, and neuronal tissue. Furthermore, TNF-*α* has been implicated in the pathogenesis of several central nervous system disorders, including cerebral ischemia, Parkinson's disease, and brain injury [10], as central mediators of tissue injury and inflammation. Extracellular TNF-*α* interacts with two cognate receptors, such as low-affinity p55 (TNFR1) and high-affinity p75 (TNFR2). Moreover, the activation of TNFR1 predominantly results in initiation of caspases involved in apoptosis [11, 12]. Thus, intracellular signaling suppression by TNF-*α* inhibition may be expected to be neuroprotective.

Etanercept (ETN) is a completely human fusion TNF-soluble receptor that inhibits the effect of the proinflammatory cytokine TNF-*α*, which has an important role in synovitis and joint damage in rheumatoid arthritis (RA) [13]. ETN acts as a decoy receptor by binding to TNF-*α* and TNF-*β*. ETN has been approved for treatment of RA in Japan since 2005 to reduce the biological activity of TNF by inhibiting the interaction between TNF receptors and TNF, and a marked effect on RA has been observed. In contrast, patients with ischemic stroke or diabetes have been recognized to show higher plasma concentrations of TNF-*α*, which is caused by elevated inflammation. Moreover, it has been reported that TNF-*α* concentration is increased in the cerebrospinal fluid in the ischemia. In the previous report, the traumatic brain injury- (TBI-) induced overproduction of IL-1*β*, TNF-*α*, and IL-6 in serum was significantly reduced by anti-TNF-*α* blockers. In contrast, etanercept therapy significantly increased the serum levels of IL-10 during TBI in rats. Furthermore, inhibition of gliosis has been observed in the brain [14]. In addition, NMDA receptor antagonist (MK801) and dexmedetomidine treatment has been reported to inhibit the production of TNF-*α* and improve cerebral infarction in the MCAO model [15, 16]. In recent years, inflammatory markers have been attracting attention as potential diagnostic markers [17, 18]. Therefore, the inflammatory reactions occurring in ischemic brain damage have increased interest in the development of therapies.

The objective of this study was to determine whether ETN-induced inhibition of TNF-*α* biological activity could improve brain damage caused by cerebral ischemia in streptozotocin- (STZ-) induced diabetic rats.

## 2. Materials and Methods

### 2.1. Animals and Reagents

Male Sprague-Dawley rats (4 weeks old, weight 120–140 g) were purchased from Japan SLC (Shizuoka, Japan) and housed under standard conditions in a temperature-controlled environment (23°C ± 0.5°C) with a cycle of 12 h of light and 12 h of darkness. The animals were allowed free access to rodent chow (CE-2; CLEA Japan, Tokyo, Japan) and water. Type 1 diabetes was induced in the rats by a single intraperitoneal injection of STZ (Sigma-Aldrich, St. Louis, MO, USA) (50 mg/kg of body weight) dissolved in 0.1 mM sodium citrate, pH 4.5 (diabetic; DM group), and the normal control rats (nondiabetic; non-DM group) were injected with the buffer only [6, 19]. Seven days after the injection of STZ, a blood sample was collected by tail vein paracentesis, and then plasma glucose was measured using a glucose analyzer (Ascensia; Bayer Yakuhin, Osaka, Japan). Diabetes was defined as a blood glucose level >300 mg/dL. Following this, the DM and non-DM groups were divided into two groups each, and the rats were housed for additional 6 weeks until stroke was induced by middle cerebral artery occlusion/reperfusion (MCAO/Re). Animal care and surgical procedures were performed in accordance with the guidelines approved by the National Institutes of Health (USA) and the Josai University Animal Research Committee. ETN was purchased from Pfizer Japan Inc. (Tokyo, Japan). The rats subjected to MCAO were divided into six treatment groups: Treatment 1, where non-DM rats were treated with ETN (300, 450, and 900 *μ*g/kg, i.p.) within 24 h before MCAO, Treatment 2, where non-DM rats were treated with ETN (300, 450, and 900 *μ*g/kg, i.v.) immediately after MCAO, Treatment 3, where non-DM rats were treated with ETN (300, 450, and 900 *μ*g/kg, i.v.) immediately after MCAO/Re, Treatment 4, where DM rats were treated with ETN (450 and 900 *μ*g/kg, i.v.) within 24 h before MCAO, Treatment 5, where DM rats were treated with ETN (450 and 900 *μ*g/kg, i.v.) immediately after MCAO, and Treatment 6, where ETN (450 or 900 *μ*g/kg, twice/week, i.p.) was repeatedly administered after the onset of diabetes for 5 weeks.

### 2.2. Middle Cerebral Artery Occlusion/Reperfusion

The experimental MCAO/Re rat model was prepared as described previously [6, 19]. The rats were anesthetized with 4% halothane and maintained with 1.5% halothane and 30% oxygen under spontaneous respiration. After a midline incision on the neck, the right common carotid artery was exfoliated under an operating microscope. All of the branches of the external carotid artery were ligated and isolated. The tip of the 4-0 surgical nylon monofilament rounded by flame heating was inserted through the internal carotid artery. When mild resistance was felt, the insertion was stopped. After occlusion for 2 h, the filament was withdrawn to enable reperfusion. The distance from bifurcation of the common carotid artery to the tip of the suture was approximately 20 mm in all of the rats. Cerebral blood flow was measured by laser Doppler flowmetry (ATBF-LC1; Unique Medical, Tokyo, Japan), and approximately 50% reduction of the baseline flow rate associated with MCAO was established in the non-DM and DM rats. The rats were allowed to recover from anesthesia at room temperature. The rectal temperature was maintained at 37°C using a heat lamp and a heating pad during the operation. All of the rats were killed after 24 h of reperfusion. A sham (control) operation involved the same manipulations but without insertion of the monofilament.

### 2.3. Plasma TNF-*α* Concentration

Enzyme-linked immunosorbent assay (ELISA) kits (Shibayagi, Gunma, Japan) were used according to the manufacturer's instructions to determine the secretion of TNF-*α* in plasma.

### 2.4. Infarction Assessment

After 24 h of reperfusion, the rats were subjected to general anesthesia using halothane and then decapitated. The brain was immediately removed and placed in ice-cold saline. Each brain was then placed in a brain matrix, and coronal sections were cut into 2 mm slices. The brain slices were immediately immersed in 2% 2,3,5-triphenyl tetrazolium chloride (TTC) (Wako Pure Chemicals Industries, Osaka, Japan) at 37°C for 15 min and then in 4% formaldehyde [19, 20]. Following this, infarction areas were identified by an image analysis system (Scion Image 1.62; Scion Corporation, Frederick, MD, USA) and were combined to obtain the infarction volumes per brain according to the following formula: corrected infarction volume (%) = [left hemisphere volume − (right hemisphere volume − the infarction volume)] × 100/left hemisphere volume.

### 2.5. Neurological Evaluation

Postischemic neurological deficits were evaluated after 24 h of reperfusion on a five-point scale as follows: grade 0, no deficit; grade 1, failure to fully extend the right forepaw; grade 2, spontaneous circling or walking to a contralateral side; grade 3, walking only when stimulated; grade 4, not responding to stimulation and a depressed level of consciousness; and grade 5, death [19, 20]. Before MCAO, the neurological score was zero in all rats. The rats that did not exhibit neurological deficits after MCAO/Re were excluded from the study. Scoring was performed blindly on individual animals and averaged in groups.

### 2.6. Terminal Deoxyribonucleotidyl Transferase-Mediated Biotin-16-dUTP Nick End-Labeling Staining

Apoptosis in the brain tissues was measured by the Apoptosis In Situ Detection Kit Wako (Wako Pure Chemicals Industries), which is based on the terminal deoxyribonucleotidyl transferase-mediated biotin-16-dUTP nick end-labeling (TUNEL) procedure and involves addition of fluorescein-deoxyuridine triphosphate to 3′-terminals of apoptotically fragmented DNA with terminal deoxynucleotidyl transferase followed by immunochemical detection using anti-fluorescein antibody conjugated with horseradish peroxidase and 3-3′-diaminobenzidine tetrachloride as a substrate. Coronal brain sections (8 *μ*m thick) were used for the assay. The slides were lightly counterstained with hematoxylin and observed under a microscope (BX51W1; Olympus, Tokyo, Japan). Quantification of TUNEL-positive cells was achieved by cell counting in areas of the penumbral cortex affected by ischemia. Three randomly chosen visual fields were counted in each region by an investigator without knowledge of the experimental conditions. The percentage of apoptotic cells was calculated by the apoptosis index, that is, dividing the number of positive-stained nuclei by the total number of nuclei [8].

### 2.7. Immunohistochemistry

Immunohistochemical staining was performed as described previously [21, 22]. The brain was fixed with 4% phosphate-buffered paraformaldehyde. Coronal brain sections (8 *μ*m thick) were incubated with 3% hydrogen peroxide for 40 min at room temperature to inhibit endogenous peroxidase and then incubated with blocking buffer (4% Block Ace; Dainippon Sumitomo Pharma, Osaka, Japan) for 2 h. Following this, the slices were incubated with polyclonal rabbit anti-TNF-*α* antibody (1 : 200; Hycult Biotech, PB Uden, Netherlands) and monoclonal mouse anti-myeloperoxidase (MPO) antibody (1 : 100, Hycult Biotech) in 10 mmol/L phosphate-buffered saline (PBS) overnight at 4°C. After washing with PBS, the slices were incubated with either Cy3-conjugated donkey anti-rabbit IgG antibody (1 : 200; Millipore, Billerica, MA, USA) or FITC-conjugated goat anti-mouse IgG antibody (1 : 100; Zymed Laboratories, San Francisco, CA, USA) at room temperature for 2 h. Finally, the sections were incubated with the nuclear stain TO-PRO-3 (1 : 10,000; Invitrogen, Carlsbad, CA, USA) in PBS for 10 min at room temperature with gentle agitation, washed, and mounted using a 70% glycerol mounting medium. Immunofluorescence was visualized by a laser scanning confocal microscope (FluoView FV1000; Olympus). Fluorescence intensity was measured by imaging software (FV10-ASW 1.7; Olympus). Analyses of immunohistochemistry were performed by an investigator blinded to the treatment protocol. Three sections per rat and three to four rats per group were used for the analyses.

### 2.8. Statistical Analysis

The data are presented as mean ± SD. Two-way ANOVA and the subsequent post hoc Tukey's multiple comparison test were used for statistical analysis. Neurological deficit scores were analyzed by performing Kruskal-Wallis test followed by Mann-Whitney *U* test. In all cases, a *P* value of < 0.05 was assumed to denote statistical significance.

## 3. Results

### 3.1. Blood Glucose and Body Weight

Body weight and blood glucose data from the experimental rats were obtained throughout the study period (Table 1). The DM group had significantly decreased body weights and increased blood glucose levels relative to those of the non-DM control group. There were no significant differences in these parameters between the ETN-treated groups and their controls (data not shown).

### 3.2. Temporal Change in Plasma TNF-*α* Levels after Transient MCAO with Reperfusion

**Figure 1:**
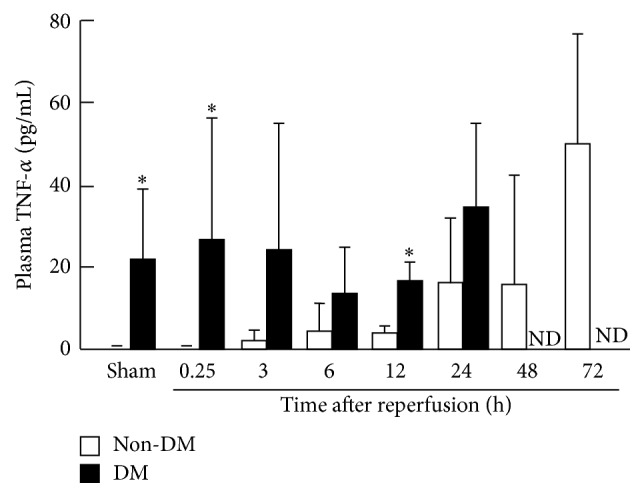
TNF-*α* concentrations in plasma after cerebral ischemia. The quantity of TNF-*α* in plasma after ischemia was determined by enzyme-linked immunosorbent assay. The open column is the non-DM group, and the closed column is the DM group. Data are means ± SDs (*n* = 4-5 per time point). ^*∗*^ *P* < 0.05 versus corresponding values for non-DM. DM, diabetes mellitus; ND, not determined; TNF, tumor necrosis factor.

The plasma levels of TNF-*α* in the non-DM and DM groups were measured by ELISA (Figure 1). The concentration of TNF-*α* was gradually increased after reperfusion in the non-DM rats. In contrast, the amount of TNF-*α* was significantly increased (about 40-fold) in the sham-operated DM rats relative to that in the sham-operated non-DM rats. Furthermore, in the DM rats, TNF-*α* remained constant after reperfusion. No difference in the concentration of TNF-*α* in the DM rats was observed between sham-operation rats and after-reperfusion rats.

### 3.3. Infarction Volume after Transient MCAO with Reperfusion

**Figure 2:**
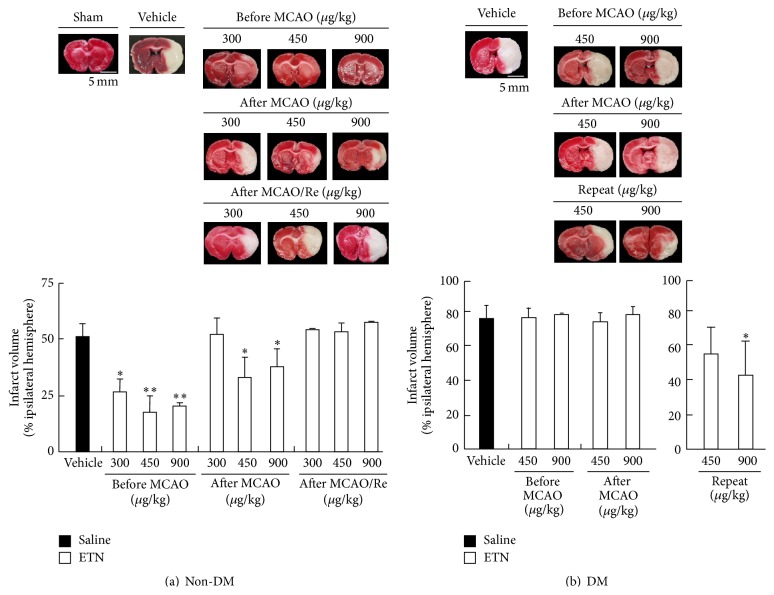
Effects of ETN on infarction induced by cerebral ischemia in non-DM and DM rat brains. (a) Representative coronal brain sections of the non-DM rats and the ETN group stained by TTC for various ETN administration conditions after reperfusion. (b) Representative coronal brain sections of the DM rats and the ETN group stained by TTC under various ETN administration conditions after reperfusion. The closed and open columns are the vehicle and ETN groups, respectively. Scale bar = 5 mm. Data are the means ± SDs (*n* = 3–6 per time point). ^*∗*, *∗∗*^ *P* < 0.05 and 0.01 for statistical significance compared with the vehicle group. DM, diabetes mellitus; ETN, etanercept; TTC, 2,3,5-triphenyl tetrazolium chloride.

Figure 2 shows representative coronal brain sections of the non-DM and DM rats stained by TTC after various ETN treatments and after or before cerebral ischemia. In the sham-operated rats, there was no apparent damage in any brain region. The infarction area in the non-DM after MCAO with 24 h reperfusion (vehicle-treated) rats was extended to the corpus striatum and cortex, whereas it was significantly decreased by ETN treatment (all groups within 24 h before MCAO or at 450 *μ*g/kg ETN immediately after MCAO). In contrast, ETN administration to rats immediately after MCAO/Re did not improve brain damage (Figure 2(a)). Because the improvement effect was not observed for administration immediately after MCAO/Re in the non-DM group, this condition was not examined in the DM group. Instead, because increased expression of TNF-*α* was already observed in the sham DM group, a new group was prepared which received repeated ETN doses immediately after the onset of diabetes. Brain injury induced by MCAO/Re was remarkably exacerbated by DM state. In contrast, reduction of infarction was not observed in the single-dose group within 24 h before MCAO/Re or immediately after MCAO in the DM rats. However, repeated administration of 450 *μ*g/kg ETN led to a decreasing trend of infarction. In addition, significant improvement was clearly shown by the 900 *μ*g/kg dose (Figure 2(b)). The timing of administration of saline had no effect (data not shown).

### 3.4. Neurological Deficits after Transient MCAO with Reperfusion

**Figure 3:**
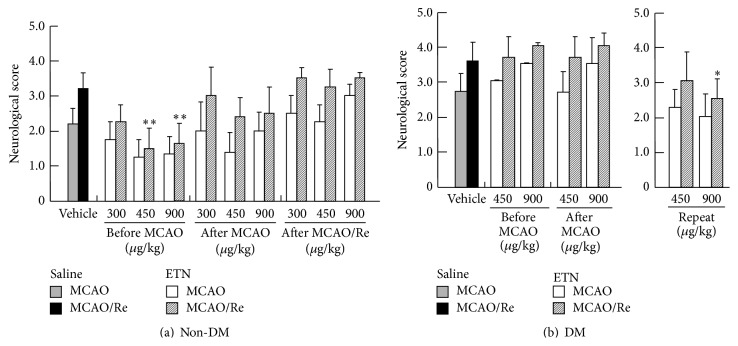
Effects of ETN on neurological deficits induced by cerebral ischemia in non-DM and DM rats. Assessment of neuronal damage after ischemia in non-DM (a) and DM (b) rats was determined by neurological score. Postischemic neurological deficits were evaluated at 2 h of MCAO and various ETN administration conditions after reperfusion. The shaded column is the MCAO-vehicle group; the closed column is the MCAO/Re-vehicle group; the open column is the MCAO-ETN group; the hatched column is the MCAO/Re-ETN group. Data are the means ± SDs (*n* = 3–6). ^*∗*, *∗∗*^ *P* < 0.05 and 0.01 versus corresponding values for the MCAO/Re-vehicle group. DM, diabetes mellitus; ETN, etanercept; MCAO/Re, middle cerebral artery occlusion/reperfusion.

MCAO for 2 h in rats resulted in moderate neurological deficits, and the neurological evaluation value was increased (Figure 3). However, the ETN-pretreated (within 24 h before MCAO) non-DM rats showed significant alleviation in the neurological deficits relative to that in the vehicle-treated non-DM rats. In contrast, in the DM rats subjected to transient MCAO, severe neurological dysfunction was observed relative to that in the non-DM rats. In addition, ETN effect was not observed in the preischemic (within 24 h before MCAO) and immediately after MCAO-treatment rats. However, ETN-repeated treatment showed a significant improvement effect on neurological dysfunction caused by MCAO with reperfusion in diabetic rats. These results were consistent with those of the cerebral infarction volume.

### 3.5. Apoptosis Evaluation after MCAO with Reperfusion

**Figure 4:**
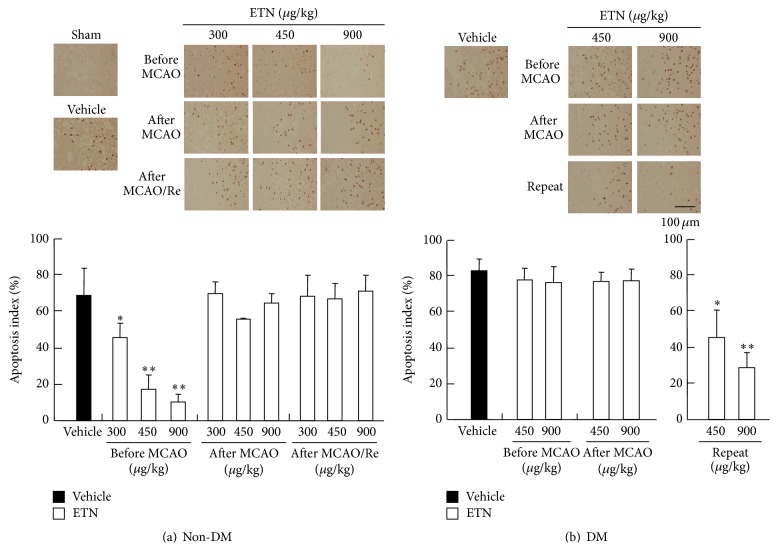
Effects of ETN on neuronal apoptosis induced by cerebral ischemia in rat brains. Representative photomicrograph and apoptosis index showing terminal deoxyribonucleotidyl transferase-mediated biotin-16-dUTP nick end-labeling-positive cells in the penumbra cortex in the non-DM group (a) and DM group (b) treated with ETN before and after ischemia. The closed column is the vehicle group, and the open column is the ETN group. Scale bar = 100 *μ*m. Data are the means ± SDs (*n* = 3–7). ^*∗*, *∗∗*^ *P* < 0.05 and 0.01 for statistical significance compared with the vehicle group. DM, diabetes mellitus; ETN, etanercept.

Representative histological images of TUNEL staining in the non-DM vehicle, ETN-pretreated non-DM, DM vehicle, and ETN-pretreated DM groups subjected to MCAO and 24 h reperfusion are shown in Figure 4. Similar to the results of TTC staining, the number of TUNEL-positive cells was remarkably increased by MCAO/Re in the DM vehicle group relative to that in the non-DM vehicle group. Pretreatment of ETN (300, 450, and 900 *μ*g/kg) significantly inhibited apoptosis activation induced by MCAO/Re in the non-DM groups. In contrast, ischemia treatment after administration of ETN (450 and 900 *μ*g/kg) did not inhibit apoptosis in the DM rats. However, the DM rats were remarkably suppressed by repeated administration of ETN (450 and 900 *μ*g/kg).

### 3.6. Inflammatory Activity after Transient MCAO with Reperfusion

**Figure 5:**
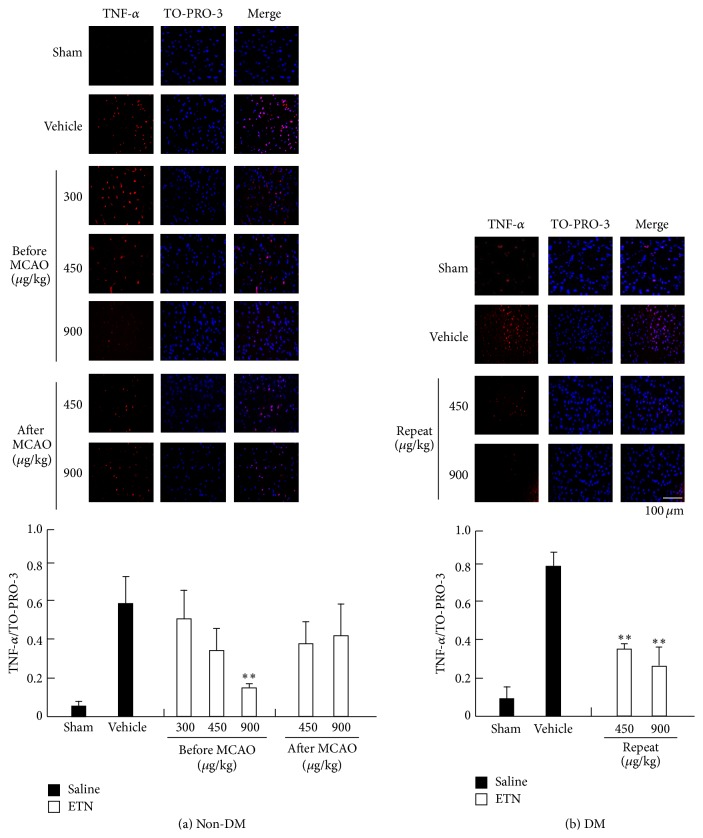
Immunohistochemical study of TNF-*α* in the cortex. Detection of TNF-*α* in the sham-operated non-DM (a) and DM (b) rat cortical neurons was determined by immunostaining and confocal imaging. The closed column is the sham or vehicle group, and the open column is the ETN group. Scale bar = 100 *μ*m. ^*∗∗*^ *P* < 0.01 for statistical significance compared with the vehicle group. DM, diabetes mellitus; TNF, tumor necrosis factor.

**Figure 6:**
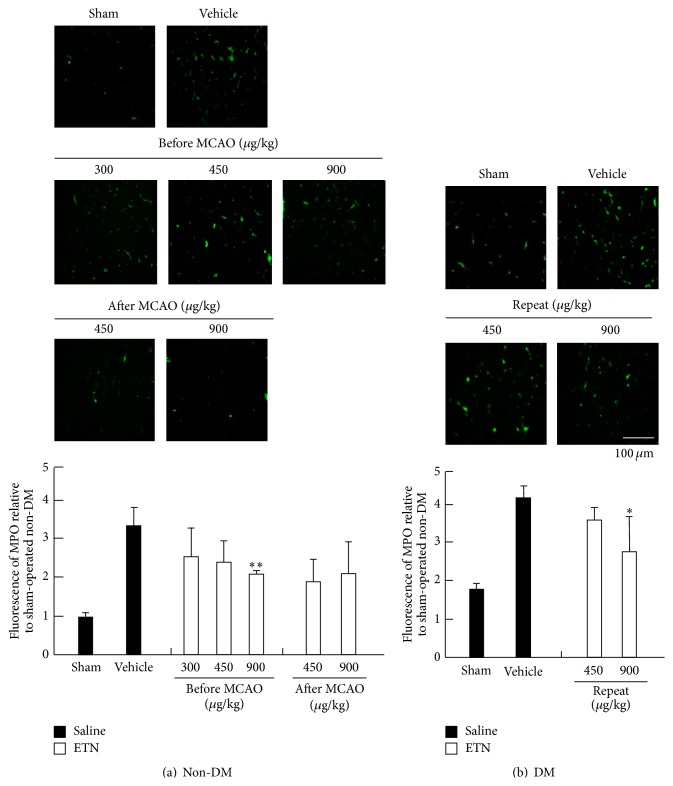
Immunohistochemical study of MPO activity in the cortex. Detection of MPO activity in the sham-operated non-DM (a) and DM (b) rat cortical neurons was determined by immunostaining and confocal imaging. The closed column is the sham or vehicle group, and the open column is the ETN group. Scale bar = 100 *μ*m. ^*∗*, *∗∗*^ *P* < 0.05 and 0.01 for statistical significance compared with the vehicle group. DM, diabetes mellitus; MPO, myeloperoxidase.

To assess the effects of ETN treatment on expression of inflammatory factors, we performed immunohistochemical staining for TNF-*α* and MPO activities (Figure 5). The effect of expression of TNF-*α* in the brain cortex penumbra on infarction was evaluated by TTC staining in the non-DM and ischemia-treatment groups. Expression of TNF-*α* was reduced in a dose-dependent manner in the previous ETN treatment group. Furthermore, non-DM rats that were injected with ETN immediately after MCAO showed improvements at both concentrations of ETN (450 and 900 *μ*g/kg). In contrast, the sham-operated DM rats had an increased number of TNF-*α*-positive cells. Expression of TNF-*α* was remarkably suppressed by repeated administration of ETN (450 and 900 *μ*g/kg) in the DM rats. To examine the effect of ETN on the leukocytic infiltration, we investigated the expression of MPO (Figure 6). The MPO activity was decreased in a dose-dependent manner of ETN by the administration within 24 h before MCAO in the non-DM group. On the other hand, the MPO activity in the cortex that was increased during ischemia was enhanced in the DM group relative to that in the non-DM group. However, this activity was significantly suppressed by repeated administration of ETN from immediately after the onset of diabetes.

## 4. Discussion

Presence of diabetes is a risk factor for exacerbation of acute cerebral ischemic injury after cerebral infarction. In previous studies, we demonstrated that STZ-induced diabetic state markedly aggravated MCAO/Re-induced neurological deficits, infarction, and apoptosis in the rat brain [21]. Furthermore, we showed that levels of superoxide generation and proinflammatory cytokines (TNF-*α* and IL-1*β*) were upregulated in the DM cortex and were remarkably enhanced during reperfusion after ischemia [21, 22]. Therefore, we have assumed that the inflammatory response aggravates ischemic brain damage. The results of microarray analysis by IL-1*β* treatment of primary cultured astrocytes have been reported to give a change in the expression of 1,400 genes such as cytokines, chemokines, and matrix metalloproteinases (MMP) [16, 23]. In addition, TNF-*α* and IL-1*β* activate the NF-*κ*B pathway, producing inflammatory materials such as IL-6. On the other hand, TNF-*α* and IL-1*β* are also known to promote release and production of neuroprotective factors. Therefore, the balance of the released inflammatory cytokines and anti-inflammatory cytokines will affect the subsequent cell failure [23, 24].

Blocking TNF-*α* has been proven to reduce brain damage and is considered to provide neuroprotective effects [25]. However, it is not clear if brain damage caused by cerebral infarctions exacerbated by diabetes is reduced by blocking TNF-*α*. We determined whether ETN-induced inhibition of TNF-*α*, which is upstream in the inflammatory response pathway, could provide protective effects against brain damage.

TNF-*α* is a proinflammatory cytokine that is synthesized in the brain within 1 h of an acute experimental ischemic stroke [26]. Intracerebroventricular injection of TNF-*α* exacerbates the extent of infarctions in experimental stroke [27]. Recent studies have reported that ETN suppressed brain injuries, such as cerebral contusions and subarachnoid hemorrhages [14, 28, 29]. Therefore, anti-TNF-*α* blockers such as ETN are expected to suppress aggravation of brain injury in diabetes. Measurements of blood TNF-*α* levels in rats with diabetes and ischemia showed that TNF-*α* increased with elapsed time after ischemia in the non-DM group. In contrast, TNF-*α* plasma concentrations in the DM group were about 40 times higher than those in the non-DM group. Thus, the diabetes pathology of chronic inflammatory reaction may have been enhanced in the whole body. Furthermore, because the plasma concentration of TNF-*α* was increased after cerebral ischemia, we assessed the cerebroprotective effect of ETN. In addition, to elucidate the effectiveness of administration of the drug, we examined the dose and timing of administration. We also examined the effectiveness of administration before and after treatment of cerebral ischemia. When ETN was intravenously administered after reperfusion in the non-DM rats, a cerebroprotective effect was not observed at any dose. However, improvement was observed at doses >450 *μ*g/kg after ischemia. In addition, the non-DM group showed significantly decreased infarction size in all groups which were injected with ETN within 24 h before ischemia. In contrast, the DM group did not show any reduction of infarction size under all conditions. Therefore, we tried repeated administration of ETN for the purpose of inhibiting TNF-*α* in DM rats. A cerebroprotective effect was observed after repeated administration of ETN (900 *μ*g/kg) immediately after diabetes onset. As shown in Figure 1, the amount of TNF-*α* in the sham-operation DM group was significantly increased relative to that in the non-DM group. Therefore, the cerebroprotective effect may depend on an effective ETN dose level. Activation of TNF-*α* signaling has been reported to induce apoptosis [30–33]. Moreover, TNF-*α* was shown to promote expression of MMP-9 and ICAM-1, induce infiltration of cells, and induce destruction of the blood-brain barrier (BBB) [34–36]. Furthermore, we confirmed that expression of TNF-*α* infiltration in leukocytes and apoptosis was observed after ischemia. However, ETN administration significantly inhibited inflammation and apoptosis. Expression of TNF receptors, such as TNFR1, was found to be elevated in the brain cortex in the DM and non-DM groups after ischemia (data not shown here). Therefore, diabetes, which may promote intracellular signaling by interaction with TNF receptors, may have enhanced inflammatory response.

For a drug to act in the brain, it is necessary to consider the permeability of the BBB. We do not have any data for the transition rate of ETN into the brain at present. Macromolecules in the blood are known to migrate to the brain from the results of permeation experiments after ischemia, as shown by Evans blue staining [22, 37, 38]. Consequently, ETN might have passed through to brain by the BBB failure after ischemia. The reason that there was no effect of the drug in the DM rats, compared with non-DM rats, may be because of inflammation and nerve damage caused by ischemia. Recent evidence suggests that high-mobility group box 1 (HMGB1) prompted induction of proinflammatory mediators, including TNF-*α*, IL-1*β*, and cyclooxygenase-2 (COX-2), and contributed to postischemic brain damage [39–41]. In a previous study, we demonstrated that HMGB1 was released from necrotic cells in the early stage of ischemia in DM rats compared with non-DM rats [42]. Moreover, a COX inhibitor significantly attenuated TNF-*α*-induced BBB breakdown and free radical formation, which indicated that MMP-mediated BBB disruption during neuroinflammation can be significantly reduced by administration of COX inhibitors [43, 44]. It has been reported that the various cytokines causing ischemic brain disorders participate in complicated ways [9, 45]. Furthermore, TNF-*α*, which is involved in inflammation and cell injury, may affect the efficacy of a drug through two different activities that protect cells through TNFR2 [11, 12, 46]. To elucidate the details of ischemic injury in DM rats, further analysis is necessary in the future.

These results suggested that repeated administration of ETN relieves exacerbation of cerebral ischemic injury in diabetic rats primarily by its anti-inflammatory effects.

## 5. Conclusions

Our study results showed that inhibition of TNF-*α* by repeated ETN administration resulted in significant reduction of inflammation and neuronal cell death after experimentally induced cerebral ischemic brain injury in DM rats.

## Figures and Tables

**Table 1:** Body weight and blood glucose levels in the non-DM and DM groups of rats.

Group	Body weight (g)	Blood glucose (mg/dL)
Non-DM	364 ± 27	123 ± 11
DM	261 ± 29^**∗∗**^	423 ± 47^**∗∗**^

The data are the means ± SD.

^**∗∗**^ *P* < 0.01 versus the non-DM group (*n* = 60).

DM, diabetes mellitus (*n* = 60).

## References

[1] Stephens J. W., Khanolkar M. P., Bain S. C. (2009). The biological relevance and measurement of plasma markers of oxidative stress in diabetes and cardiovascular disease. *Atherosclerosis*.

[2] Baynes J. W. (1991). Role of oxidative stress in development of complications in diabetes. *Diabetes*.

[3] Vinik A., Flemmer M. J. (2002). Diabetes and macrovascular disease. *Journal of Diabetes and its Complications*.

[4] Biller J., Love B. B. (1993). Diabetes and stroke. *The Medical Clinics of North America*.

[5] Ahn T., Yun C.-H., Oh D.-B. (2006). Tissue-specific effect of ascorbic acid supplementation on the expression of cytochrome P450 2E1 and oxidative stress in streptozotocin-induced diabetic rats. *Toxicology Letters*.

[6] Iwata N., Okazaki M., Kasahara C. (2008). Protective effects of a water-soluble extract from culture medium of *Ganoderma lucidum* mycelia against neuronal damage after cerebral ischemia/reperfusion in diabetic rats. *Journal of Japan Society of Nutrition and Food Science*.

[7] Rizk N. N., Rafols J., Dunbar J. C. (2005). Cerebral ischemia induced apoptosis and necrosis in normal and diabetic rats. *Brain Research*.

[8] Li Z.-G., Britton M., Sima A. A. F., Dunbar J. C. (2004). Diabetes enhances apoptosis induced by cerebral ischemia. *Life Sciences*.

[9] Amantea D., Nappi G., Bernardi G., Bagetta G., Corasaniti M. T. (2009). Post-ischemic brain damage: pathophysiology and role of inflammatory mediators. *The FEBS Journal*.

[10] Scherbel U., Raghupathi R., Nakamura M. (1999). Differential acute and chronic responses of tumor necrosis factor-deficient mice to experimental brain injury. *Proceedings of the National Academy of Sciences of the United States of America*.

[11] Watters O., O'Connor J. J. (2011). A role for tumor necrosis factor-*α* in ischemia and ischemic preconditioning. *Journal of Neuroinflammation*.

[12] Park K. M., Bowers W. J. (2010). Tumor necrosis factor-alpha mediated signaling in neuronal homeostasis and dysfunction. *Cellular Signalling*.

[13] Takeuchi T., Miyasaka N., Kawai S. (2015). Pharmacokinetics, efficacy and safety profiles of etanercept monotherapy in Japanese patients with rheumatoid arthritis: review of seven clinical trials. *Modern Rheumatology*.

[14] Chio C.-C., Lin J.-W., Chang M.-W. (2010). Therapeutic evaluation of etanercept in a model of traumatic brain injury. *Journal of Neurochemistry*.

[15] Sriram K., O'Callaghan J. P. (2007). Divergent roles for tumor necrosis factor-*α* in the brain. *Journal of Neuroimmune Pharmacology*.

[16] Tanabe K., Iida H. (2013). The role of astrocytes in neuroprotection. *Anesthesia 21 Century*.

[17] Sapojnikova N., Kartvelishvili T., Asatiani N. (2014). Correlation between MMP-9 and extracellular cytokine HMGB1 in prediction of human ischemic stroke outcome. *Biochimica et Biophysica Acta: Molecular Basis of Disease*.

[18] Oozawa S., Sanoa S., Nishibori M. (2014). Usefulness of high mobility group box 1 protein as a plasma biomarker in patient with peripheral artery disease. *Acta Medica Okayama*.

[19] Iwata N., Okazaki M., Xuan M., Kamiuchi S., Matsuzaki H., Hibino Y. (2014). Orally administrated ascorbic acid suppresses neuronal damage and modifies expression of SVCT2 and GLUT1 in the brain of diabetic rats with cerebral ischemia-reperfusion. *Nutrients*.

[20] Kusaka I., Kusaka G., Zhou C. (2004). Role of AT_1_ receptors and NAD(P)H oxidase in diabetes-aggravated ischemic brain injury. *American Journal of Physiology: Heart and Circulatory Physiology*.

[21] Iwata N., Okazaki M., Nakano R., Balestrino M. (2012). Diabetes-mediated exacerbation of neuronal damage and inflammation after cerebral ischemia in rat: protective effects of water-soluble extract from culture medium of *Ganoderma lucidum* mycelia. *Advances in the Preclinical Study of Ischemic Stroke*.

[22] Wang L., Li Z., Zhang X. (2014). Protective effect of shikonin in experimental ischemic stroke: Attenuated TLR4, p-p38MAPK, NF-*κ*B, TNF-*α* and MMP-9 expression, up-regulated claudin-5 expression, ameliorated BBB permeability. *Neurochemical Research*.

[23] Allan S. M., Tyrrell P. J., Rothwell N. J. (2005). Interleukin-1 and neuronal injury. *Nature Reviews Immunology*.

[24] Zhao Y., Rempe D. A. (2010). Targeting astrocytes for stroke therapy. *Neurotherapeutics*.

[25] Arango-Dávila C. A., Vera A., Londoño A. C. (2015). Soluble or soluble/membrane TNF-*α* inhibitors protect the brain from focal ischemic injury in rats. *International Journal of Neuroscience*.

[26] Liu T., Clark R. K., McDonnell P. C. (1994). Tumor necrosis factor-alpha expression in ischemic neurons. *Stroke*.

[27] Barone F. C., Arvin B., White R. F. (1997). Tumor necrosis factor-*α*. A mediator of focal ischemic brain injury. *Stroke*.

[28] Zhang B.-F., Song J.-N., Ma X.-D. (2015). Etanercept alleviates early brain injury following experimental subarachnoid hemorrhage and the possible role of tumor necrosis factor-*α* and c-Jun n-terminal kinase pathway. *Neurochemical Research*.

[29] Tuttolomondo A., Pecoraro R., Pinto A. (2014). Studies of selective TNF inhibitors in the treatment of brain injury from stroke and trauma: a review of the evidence to date. *Drug Design, Development and Therapy*.

[30] Wang L. W., Chang Y. C., Chen S. J. (2014). TNFR1-JNK signaling is the shared pathway of neuroinflammation and neurovascular damage after LPS-sensitized hypoxic-ischemic injury in the immature brain. *Journal of Neuroinflammation*.

[31] Longhi L., Perego C., Ortolano F. (2013). Tumor necrosis factor in traumatic brain injury: effects of genetic deletion of p55 or p75 receptor. *Journal of Cerebral Blood Flow and Metabolism*.

[32] Shuh M., Bohorquez H., Loss G. E., Cohen A. J. (2013). Tumor necrosis factor-*α*: life and death of hepatocytes during liver ischemia/reperfusion injury. *Ochsner Journal*.

[33] O'Connor J. J. (2013). Targeting tumour necrosis factor-*α* in hypoxia and synaptic signalling. *Irish Journal of Medical Science*.

[34] Wei H., Wang S., Zhen L. (2015). Resveratrol attenuates the blood-brain barrier dysfunction by regulation of the MMP-9/TIMP-1 balance after cerebral ischemia reperfusion in rats. *Journal of Molecular Neuroscience*.

[35] Takata F., Dohgu S., Matsumoto J. (2011). Brain pericytes among cells constituting the blood-brain barrier are highly sensitive to tumor necrosis factor-*α*, releasing matrix metalloproteinase-9 and migrating in vitro. *Journal of Neuroinflammation*.

[36] Yang G.-Y., Gong C., Qin Z., Ye W., Mao Y., Bertz A. L. (1998). Inhibition of TNF*α* attenuates infarct volume and ICAM-1 expression in ischemic mouse brain. *NeuroReport*.

[37] Wu L., Zhang K., Hu G., Yang H., Xie C., Wu X. (2014). Inflammatory response and neuronal necrosis in rats with cerebral ischemia. *Neural Regeneration Research*.

[38] Kim G. W., Gasche Y., Grzeschik S., Copin J.-C., Maier C. M., Chan P. H. (2003). Neurodegeneration in striatum induced by the mitochondrial toxin 3-nitropropionic acid: role of matrix metalloproteinase-9 in early blood-brain barrier disruption?. *Journal of Neuroscience*.

[39] Yang Q.-W., Wang J.-Z., Li J.-C. (2010). High-mobility group protein box-1 and its relevance to cerebral ischemia. *Journal of Cerebral Blood Flow and Metabolism*.

[40] Qiu J., Nishimura M., Wang Y. (2008). Early release of HMGB-1 from neurons after the onset of brain ischemia. *Journal of Cerebral Blood Flow and Metabolism*.

[41] Pizzi M., Sarnico I., Lanzillotta A., Battistin L., Spano P. (2009). Post-ischemic brain damage: NF-*κ*B dimer heterogeneity as a molecular determinant of neuron vulnerability. *The FEBS Journal*.

[42] Iwata N., Okazaki M., Kamiuchi S. (2015). Early release of HMGB1 may aggravate neuronal damage after transient focal ischemia in diabetic rat brain. *International Journal of Diabetes and Clinical Research*.

[43] Candelario-Jalil E., Yang Y., Rosenberg G. A. (2009). Diverse roles of matrix metalloproteinases and tissue inhibitors of metalloproteinases in neuroinflammation and cerebral ischemia. *Neuroscience*.

[44] Candelario-Jalil E., Taheri S., Yang Y. (2007). Cyclooxygenase inhibition limits blood-brain barrier disruption following intracerebral injection of tumor necrosis factor-*α* in the rat. *The Journal of Pharmacology and Experimental Therapeutics*.

[45] Suzuki S., Tanaka K., Suzuki N. (2009). Ambivalent aspects of interleukin-6 in cerebral ischemia: inflammatory versus neurotrophic aspects. *Journal of Cerebral Blood Flow and Metabolism*.

[46] Hallenbeck J. M. (2002). The many faces of tumor necrosis factor in stroke. *Nature Medicine*.

